# “Black Women Should Not Die Giving Life”: The lived experiences of Black women diagnosed with severe maternal morbidity in the United States

**DOI:** 10.1111/birt.12820

**Published:** 2024-04-02

**Authors:** Wendy Post, Angela Thomas, Karey M. Sutton

**Affiliations:** ^1^ Georgetown University Washington DC USA; ^2^ Medstar Research Institute Hyattsville Maryland USA

**Keywords:** birth, black women, severe maternal morbidity

## Abstract

**Objective:**

We sought to understand the lived experiences of Black women diagnosed with severe maternal morbidity (SMM) in communities with high maternal mortality to inform practices that reduce obstetric racism and improve patient outcomes.

**Methods:**

From August 2022 through December 2022, we conducted a phenomenological, qualitative study among Black women who experienced SMM. Participants were recruited via social media and met inclusion criteria if they self‐identified as Black cisgender women, were 18–40 years old, had SMM diagnosed, and lived within zip codes in the United States that have the top‐five highest maternal mortality rates. Family members participated on behalf of women who were deceased but otherwise met all other criteria. We conducted in‐depth interviews (IDIs), and transcripts were analyzed using inductive and deductive methods to explore birth story experiences.

**Results:**

Overall, 12 participants completed IDIs; 10 were women who experienced SMM and 2 were mothers of women who died due to SMM. The mean age for women who experienced SMM was 31 years (range 26–36 years) at the time of the IDI or death. Most participants had graduate‐level education, and the average annual household income was 123,750 USD. Women were especially interested in study participation because of their high‐income status as they did not fit the stereotypical profile of Black women who experience racial discrimination. The average time since SMM diagnosis was 2 years. Participants highlighted concrete examples of communication failures, stereotyping by providers, differential treatment, and medical errors which patients experienced as manifestations of racism. Medical personnel dismissing and ignoring concerns during emergent situations, even when raised through strong self‐advocacy, was a key factor in racism experienced during childbirth.

**Conclusions:**

Future interventions to reduce racism and improve maternal health outcomes should center on the experiences of Black women and focus on improving patient–provider communication, as well as the quality and effectiveness of responses during emergent situations.

Précis statement: This study underscores the need to center Black women's experiences, enhance patient–provider communication, and address emergent concerns to mitigate obstetric racism and enhance maternal health outcomes.

## 1 INTRODUCTION

In some geographic locations in the United States (US), Black women experience an eightfold risk of maternal mortality compared with the national average.^1^, ^2^ These disparities persist despite controlling for factors such as socioeconomic status and education levels.^3^, ^4^ For every maternal death in the US, approximately 100 women experience a near‐fatal complication of severe maternal morbidity (SMM).^5^ SMM diagnoses are unanticipated labor and delivery outcomes resulting in short‐term and long‐term health consequences and, according to the US Centers for Disease Control and Prevention (CDC), include hemorrhage, infection, amniotic fluid embolism, thrombotic pulmonary or embolic disorders, hypertensive disorders, anesthesia complications, and cardiovascular and other non‐cardiovascular medical conditions.^6^ The Association of Women's Health, Obstetric, and Neonatal Nurses (AWHONN) addresses lower extremity nerve injury in childbirth through a practice brief, indicating its significance in the field of obstetric and neonatal nursing.^7^ SMM should be rare, and these conditions are often preventable in high‐resource settings. However, the US continues to be an outlier among high‐resource nations.^1^, ^8^, ^9^ While SMM also occurs in women of other ethnic backgrounds, these diagnoses often are most severe and deadly in Black women, who experience SMM at a rate double that of white women in the US.^10^, ^11^

While there have been persistent calls to address disparities in SMM among Black women, there is a notable scarcity of research that explores the lived experiences—personal knowledge gained through firsthand involvement—of SMM in this population. To date, studies of disparities in SMM among Black women in the US are primarily quantitative and analyze data from birth and death certificates, vital statistics, and medical chart reviews.^8^, ^12^ These data help identify the causes of maternal morbidity and mortality and detect racial and ethnic disparities.^13^ Race is not a causative factor for SMM.^14^, ^15^ Yet Black women often experience inconsistent and substandard obstetric care compared with other women due to racism, bias, and discrimination,^14^, ^16^, ^17^, ^18^ which subsequently contributes to SMM. To understand how these factors place Black women at such high risk of SMM in the US, it is crucial to engage Black women in the inquiry process and center their experiences.^13^ Storytelling is part of Black culture in the US.^19^ For individuals navigating marginalization, telling their stories offers the opportunity to engage in awareness‐raising that can benefit their communities.^20^ Interpretative phenomenological studies offer insights into how an individual, in a specific context, makes sense of a situation and could be helpful for understanding the lived experience of SMM among Black women in the US.^21^

We conducted a qualitative study using an interpretive phenomenological design to translate the lived experiences of Black women, in their own words, who were diagnosed with SMM at hospitals located in geographic areas of the US that have high maternal mortality. Our goal is to inform practices that reduce racism and improve patient outcomes in obstetric care.

In addition to existing quantitative analyses, qualitative studies provide a deeper understanding of the nuances and complexities of SMM among Black women. Recent qualitative research such as Wang et al.,^22^ Mehra et al.,^23^ Eniola et al.,^24^ and Altman et al.^25^ have begun to explore these experiences in greater depth. These studies offer invaluable insights into the peripartum care of Black women, their encounters with healthcare systems, and the intersectional challenges they face, thereby contributing significantly to our understanding of the issue.

## 2 MATERIALS AND METHODS: STUDY DESIGN AND POPULATION

**FIGURE 1:**
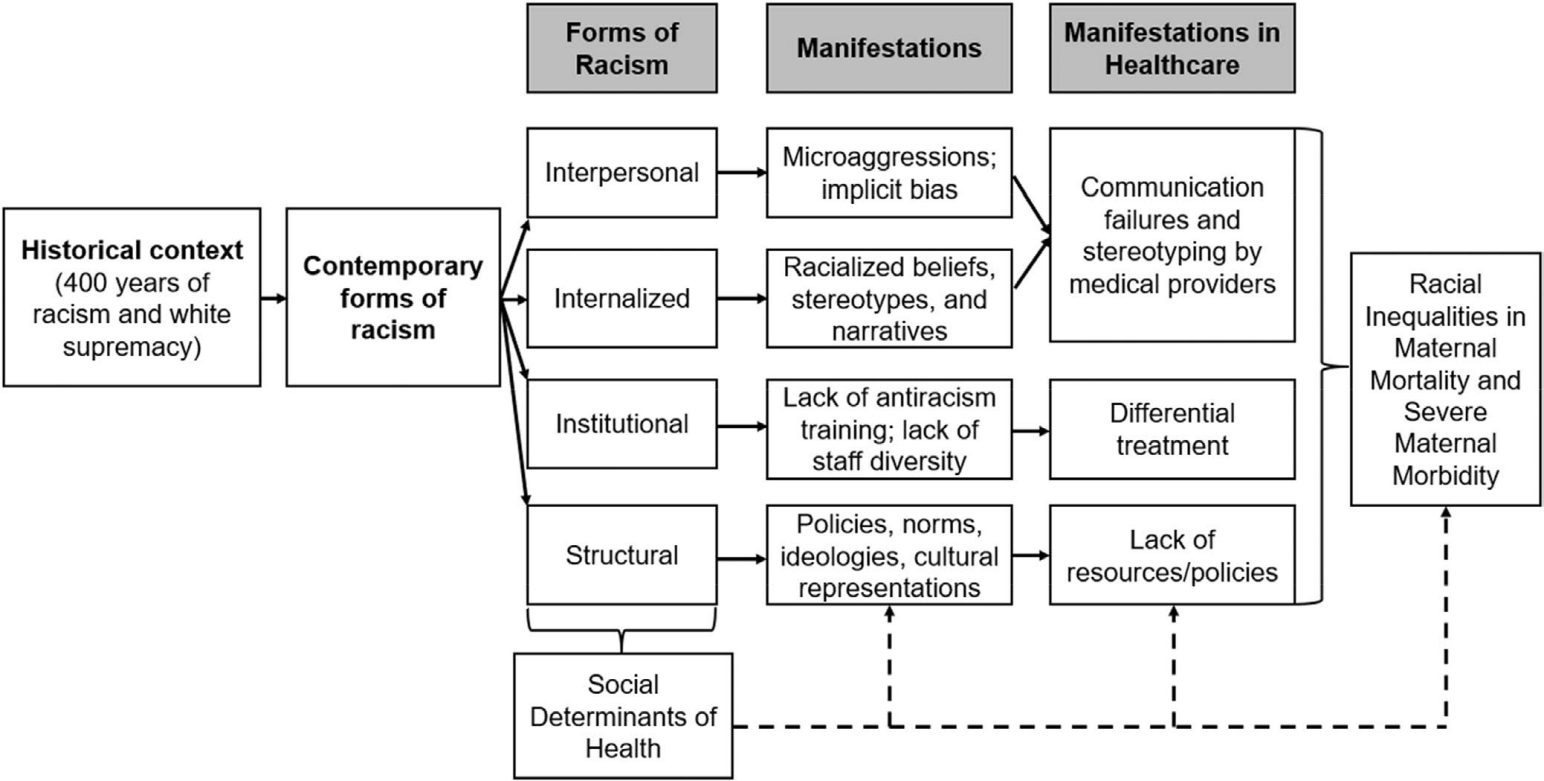
Conceptual framework adapted from the pathways to racial and ethnic disparities in severe maternal morbidity and mortality model.^20^

This qualitative study was conducted between August 2022 and December 2022 and guided by a conceptual framework (Figure 1) adapted from the Pathways to Racial and Ethnic Disparities in Severe Maternal Morbidity & Mortality model.^1^, ^22^ To select regions for participant recruitment, geographic areas were defined by zip codes and ordered by maternal mortality rates based on publicly available data.^23^ The top‐five geographic areas with the highest maternal mortality rates were selected. Participants were recruited through flyers on social media (e.g., LinkedIn, Instagram, Facebook, and TikTok), flyers at offices for the Special Supplemental Nutrition Program for Women, Infants, and Children—better known as WIC—within the selected zip codes, and referrals from earlier participants. The sample size was not predetermined but aimed to encompass a diverse range of SMM experiences, concluding when thematic saturation occurred indicating no emergence of new concepts in the data. This qualitative research approach prioritizes data depth over quantitative metrics, aligning with established methodologies recommended by Smith and Osborn (2008) and Braun and Clarke (2013). Thematic saturation ensures comprehensive coverage of the study topic.

Eligibility criteria for participation included being a Black cisgender woman, being aged 18–40 years old, and being diagnosed with one of the 21 indicators for SMM per CDC or AWHONN guidelines within 42 days of birth at a hospital in one of the following zip codes: 20060 (Washington, DC), 30303 (Atlanta, Georgia), 70112 (New Orleans, Louisiana), 46290 (Carmel, Indiana), and 10467 (Bronx, New York). The definition of “Black” included self‐identification of being from Sub‐Saharan Africa or of Afro‐diasporic descent, inclusive of the Caribbean, Central America, Sub‐Saharan Africa, Central or South America, Aboriginal Australians/Torres Strait Islanders, and Indigenous American Indian tribes (Cherokee, Chickasaw, Choctaw, Creek, and Seminole Nations). During recruitment, two mothers of eligible Black women, whose daughters had died due to SMM conditions, expressed interest in participating on behalf of their deceased daughters. The inclusion criteria were adjusted to accommodate the involvement of relatives representing deceased individuals who otherwise met all criteria. Zip codes with elevated SMM rates, notably exceeding the national average, were selected to concentrate the study on areas most affected. Language diversity, including Spanish proficiency, was taken into account to ensure broad representation within communities of color; one participant in the study was bilingual in Spanish.

### 2.1 Data collection

After providing consent, participants completed an initial questionnaire covering demographics. Subsequently, a Black cisgender woman conducted semi‐structured in‐depth interviews (IDIs) via Zoom, addressing childbirth experiences, healthcare biases, and social determinants of health. The IDI guide, shaped by literature and clinical expertise, explored themes aligned with our conceptual framework (Figure 1). Interviews, recorded and transcribed, delved into delivery experiences, preparation, and encountered biases. Reflexive Thematic Analysis principles guided our diverse research team, enhancing the study's depth by incorporating varied perspectives, including those of Black and Multiracial cisgender women and a lesbian team member.

### 2.2 Data analysis

Employing grounded theory and thematic analysis principles, the research team used Delve qualitative coding software to scrutinize and categorize transcripts. The analysis focused on themes linked to the conceptual framework, encompassing racism forms (interpersonal, internalized, institutional, and structural), intrapersonal manifestations (e.g., microaggressions, implicit bias, acceptance of racialized narratives), and healthcare‐related manifestations (communication breakdowns, provider stereotyping, differential treatment, resource/policy inadequacies, and medical errors). A codebook, developed by the primary investigator and three study staff, emerged from inductive and deductive methods applied to a subset of three transcripts. *The Georgetown Law Journal* publication entitled *Obstetric Violence* authored by Elizabeth Kakura further informed the definitions of obstetric abuse, disrespect, and coercion.^24^ Deductive codes, rooted in racism forms and conceptual framework manifestations, were complemented by inductive codes derived from multiple transcript reviews. The codebook evolved iteratively, with code definitions refined through consensus discussions. Independent coding by one team member, followed by a secondary review by another, ensured coding rigor. Discrepancies were resolved through consensus discussions. The approach combined inductive coding for participant‐centric themes and deductive coding, applying pre‐existing conceptual framework concepts. This blended approach, aligned with Braun & Clarke's methodology, fostered a dynamic and comprehensive analysis, allowing the emergence of deeply rooted themes reflective of participants' lived experiences.

### 2.3 Ethical considerations

The Georgetown‐Medstar Universities Center for Clinical and Translational Science (GHUCCTS) Institutional Review Board reviewed and approved the study protocol, informed consent forms, and data collection tools. All study participants received a $25 gift card to reimburse them for their time and were welcomed to end the interview and/or their participation at any point.

## 3 RESULTS

Overall, 15 interested potential participants responded within the recruitment timeframe; 3 did not meet the criteria because of ethnicity, zip code, or failure to meet the SMM definition. In total, 12 participants completed IDIs; 10 were women who experienced SMM and 2 were mothers of women who died due to SMM. The mean age for women who experienced SMM was 31 years (range 26–36 years) at the time of the IDI or their death (Table 1). The majority of participants identified as African American (66%); other participants identified as having descended from Sub‐Saharan Africa and/or the African diaspora, Caribbean, and Central/South American. Most participants had graduate‐level education, and the average annual household income was USD 123,750. Some participants expressed that they were especially interested in participating in the study because of their high‐income status as they felt they did not fit the stereotypical profile of Black women who experience SMM due to racial discrimination. Participants experienced various SMM diagnoses (Table 2) including uterine dehiscence, uterine rupture, hemorrhage, sepsis, eclampsia, cardiomyopathy, myocardial infarction, placental accreta, and pulmonary embolism. The average time since SMM diagnosis was 2 years; one participant had SMM nine years ago, and all other women had SMM within the last 1.5–2 years. It is noteworthy that our participant pool predominantly comprised individuals from higher income brackets. This was not a result of a targeted recruitment strategy, but rather a reflection of those who responded to our open call for participation. This demographic trend may offer a unique perspective on the intersections of race, economic status, and healthcare experiences within the context of severe maternal morbidity.

**TABLE 1:** Demographic characteristics of in‐depth interview participants (*n* = 12).

Characteristic	*N* (%) or Mean (Range)
Age (years)	31 (26–36)
**Race/ethnicity**	
African American	8 (66%)
Sub‐Saharan Africa or of the Afro‐diasporic descent	1 (8%)
Caribbean descent	1 (8%)
Central/South American descent	1 (8%)
Black American Indian tribes	1 (8%)
**Marital status**	
Single (never married)	2 (17%)
Married	8 (66%)
Divorced/separated	1 (8%)
Other	1 (8%)
**Highest completed education**	
High school	1 (8%)
Undergraduate/college	1 (8%)
Graduate school	10 (83%)
**Employment status**	
Unemployed	0 (0%)
Part‐time employed	0 (0%)
Full‐time employed	100 (100%)
**Insurance status**	
No insurance	0 (0%)
Public insurance	2 (17%)
Private insurance	10 (83%)
Gestational age at 1st prenatal visit (weeks)	15 (4)
Annual household income (US dollars)^a^	123,750 (0–375,000)

^a^ One client who was disabled reported an annual income of 0 USD.

**TABLE 2:** Obstetric characteristics of in‐depth interview participants (*n* = 12).

Participant no.	No. of prior pregnancies	Prior “high‐risk” pregnancy (yes/no)^a^	Gestational age at delivery (weeks)	Delivery method	SMM diagnoses	Level of education	Outcome
1	0	N/A	39 weeks	Emergent	Uterine dehiscence with open infection in incision; hemorrhage	College degree	ICU admission
				Cesarean	Hematological coagulation disorders		
2	0	N/A	38 weeks	Emergent	Uterine infection–endometritis; sepsis	Doctoral degree	ICU admission
				Cesarean			
3	2	No	39 weeks	Cesarean	Pulmonary embolism resulted in death^b^	Doctoral degree	Death
4	1	No	38 weeks	Vaginal delivery (assisted)	Nerve injury during childbirth, hemorrhage; pelvic neurological injury	Doctoral degree	Repeated surgical procedures‐permanent disability
5	1	Yes	39 weeks	Emergent	Eclampsia	Doctoral degree	ICU admission
				Cesarean			
6	1	No	38 weeks	Vaginal delivery (assisted)	Hemorrhage; cardiomyopathy	Doctoral degree	ICU admission
7	3	No	37 weeks	Vaginal delivery	Postpartum hemorrhage	College Degree	Re‐admission post‐discharge
8	2	No	34 weeks	Emergent	Bilateral pulmonary embolism	College Degree	ICU admission
				Cesarean			
9	0	N/A	35 weeks	Emergent	Acute myocardial infarction resulting in death^c^	Disabled—no college	Death
				Cesarean			
10	1	Yes	37 weeks	Emergent	Uterine rupture resulting in hysterectomy	College Degree	Re‐admission post‐discharge
				Cesarean			
11	2	Yes	37 weeks	Cesarean	Placental accreta resulting in complete hysterectomy	Doctoral degree	Re‐admission post discharge
12	0	N/A	39 weeks	Cesarean	Eclampsia	Doctoral degree	ICU admission

^a^ Participants were asked, “Did you have a prior pregnancy considered to be a high‐risk pregnancy?”

^b^ Autopsy found blood clots in legs and lungs; cause of death pulmonary embolism related to childbirth. Autopsy found myocardial infarction; cause of death myocardial infarction related to childbirth.

^c^ CDC SMM indicators: Acute myocardial infarction, Aneurysm, Acute renal failure, Adult respiratory distress syndrome, Amniotic fluid embolism, Cardiac arrest/ventricular fibrillation, Conversion of cardiac rhythm, Disseminated intravascular coagulation, Eclampsia, Heart failure/arrest during surgery or procedure, Puerperal cerebrovascular disorders, Pulmonary edema/Acute heart failure, Severe anesthesia complications, Sepsis, Shock, Sickle cell disease with crisis, Air and thrombotic embolism, Blood products transfusion, Hysterectomy, Temporary tracheostomy and Ventilation.^26^

Three key themes surfaced from the IDIs about experiences of SMM, all relating to the *manifestations of racism* domains in our conceptual framework: (1) communication failures and stereotyping by providers, (2) differential treatment, and (3) medical errors and near misses. The duration of these interviews varied, typically lasting around 1.5 to 2 h, which provided ample time for participants to share their comprehensive experiences.

### 3.1 Communication failures and stereotyping by providers

Participants reported multiple communication failures during childbirth which they experienced as interpersonal racism from providers, resulting in participants feeling dismissed and disrespected. Participants mentioned sharing their alarming symptoms with providers who did not address or escalate their concerns. In some cases, participants experienced emergent issues or “warning signs,” but their efforts to self‐advocate for treatment were dismissed by providers. Participants also reported that providers did not document their stated concerns which hindered subsequent clinical care since their full medical history was unknown. Participants also noted that some complications and injuries were not communicated to them and were left undocumented as explained by one participant:

“I was hemorrhaging. I lost so much blood. I needed a transfusion. I never got one. My skin, the color of my face. I was blue and I'm literally begging for help any which way I can get it. So not only do they not document my consultation, but they also did not even verbally tell me you sustained all of these complications”. [Participant #6]

Dismissiveness also manifested as a lack of prioritization and “downplaying” of symptoms, even during serious events and extreme pain. Participants felt that providers did not “believe” them, even when family members intervened to advocate on their behalf.

“We were not believed. We were not taken seriously. I relate my experience to that of [Charles Johnson, husband of Kira Johnson], he cried out, ‘Hey, my wife is in trouble.’ They told him, ‘She's not a priority’ … she is just not a priority right now … but was she a priority when she died?” [Participant #7]

Providers' failures to adequately address participants' symptoms and medical concerns when raised led to diagnostic lapses and treatment delays. In some cases, this resulted in medical emergencies and even death that could have been prevented if providers addressed concerns when they were initially raised:

“I got really sick… We went to go see a lactation specialist and I remember sweating profusely. They said, ‘You need to go to the ER. Something's wrong with you’. [At the ER] the doctor said, if I had waited any longer, I would have ended up needing a hysterectomy because I had such a bad infection from my [c‐section] surgery. I also had a dangerously high fever … I just remember feeling so scared, I thought I was going to die. Eventually they figured it out and I had something called endometritis, which is not to be confused with endometriosis. Basically, it was a really, nasty infection in my uterus.” [Participant #1]

“My daughter was autistic and non‐verbal. She was raped during an admission to one of our state's local group homes. After her death, the state coroner called persistently and encouraged me to have an autopsy done. I was reluctant. We learned that her cause of death was due to clinical negligence at the time of her delivery”. [Participant #9]

Participants felt disrespected when providers were not responsive to their concerns. In some instances, participants felt that they were not taken seriously when communicating concerns. Instead, staff were more interested in obtaining information perceived as negative stereotypes attributed Black motherhood, such as lack of resources to provide for infants and paternity issues:

“The energy from the social worker was … I bet you this black girl doesn't even have diapers at home for this baby. And I bet you she's not even prepared for this baby. And I bet you she doesn't even have a car seat … Why are we talking about diapers? I need emergency medical treatment … I'm in physical trouble. Get me an MRI—‐I told the social worker and she looked at me and said, ‘I'm here to evaluate you mentally, and I need to make sure you have diapers at home and a car seat.’ Yeah. Okay, well, that's it for my assessment … and that was the only person in the hospital that ever came to my bedside, a social worker. That was it”. [Participant #4]

“Oh—you guys aren't married? We need to make sure dad is not signing the birth certificate under duress”. [Participant #2]

These narratives illustrate the ways communication failures and stereotyping function to perpetuate discrimination, placing Black women at risk by rendering them invisible and unheard; they epitomize *communication as a discriminatory tool*.

### 3.2 Differential treatment

Participants reported receiving differential treatment based on their race. This was displayed in several ways, including being coerced into medical treatments or even receiving procedures against their expressed will. These experiences were traumatic, and at times, participants felt providers were intentionally not addressing their pain or even inflicting pain due to their vulnerable state during and after childbirth:

“The nurse came in and said, ‘You are not due for [pain] medication for another two hours.’ She made me wait. So, I literally just cried the first entire night in pain. The nurse came in the next morning and yelled at me because I had the baby in the bed with me. ‘You're not supposed to do that!’ And I responded I cannot get up to get to my baby from the bleeding and pain I am in”. [Participant #2]

“I sustained an injury to my spinal cord, to the lumbar sacral area. I sustained injury to my peripheral nerve. My pudendal nerve was traumatically injured. It was pulled at the nerve roots coming out of the lumbar sacrum area. My sciatic nerve was injured. My nerve roots at S‐2, S‐3, L4‐L‐5, and L‐1 are injured”. [Participant #4]

Many of the participants also expressed feeling taken advantage of in their vulnerable state during childbirth by providers conducting “extra” procedures to which participants did not initially consent, including surgical procedures. This led to a loss of trust in the care participants received and a perception that these extra procedures were related to the subsequent medical events:

“I don't talk about this a lot, but during her c‐section, extra things were done … things that you would have to schedule a surgery for. She had fibroids. And when they remove fibroids, you have a separate surgery for that. The doctor was acting like they were doing her a favor by removing fibroids while simultaneously giving her a c‐section. There were extra things that were done … something about that impacted her”. [Participant #3]

Additionally, participants reported experiencing negligence and neglect from providers due to racial stereotyping, despite cries for help. Participants described providers not believing them and attributing symptoms and injuries to them “being on drugs” or being “uneducated about appropriate care and treatment.” This led to severe adverse outcomes and even death:

“The ambulance came, and they kept asking, ‘Is she on drugs?’ People kept coming. The fire department. The police department. Three separate groups of people all asking the same questions. We told you she just had a baby. We told you she just had a c‐section. I'm telling you; she didn't even take the pain medication that they gave her. And I'm also telling you that I believe it's a pulmonary embolism based on the symptoms—so just check and see and do what y'all have to do. And by the time all the talking and the complaints about how she wouldn't stop moving had gone on and on, she went into cardiac arrest”. [Participant #3]

“And my husband kept pressuring the nurses, saying, ‘She's not right. She's not right’ … He kept on pressuring. It had been hours. The doctor finally comes in and checks me and his arm from above the elbow down is covered in blood. The nurses did a fundal massage, and the doctor says, ‘Oh, there seems like there's a piece of placenta left behind’ — but how do you leave behind a piece of placenta in a c‐section?” [Participant #1]

Participants' experiences with “Differential Treatment” capture the essence of inequitable treatment as a breach of trust and care; they illustrate *violations of trust as disrespectful and negligent care*.

### 3.3 Medical errors and near misses

Most participants felt that the SMM events they experienced were either directly or indirectly related to medical errors and that they constituted “near misses.” Rarely were these encounters documented by providers, and providers also did not admit their mistakes. This type of gaslighting exacerbated the birth trauma experienced by Black women, making them feel expendable, unvalued, and less than others due to the racialized treatment they received during moments of medical need. The lack of accountability on behalf of providers deepened emotional wounds precipitated by the physical trauma of childbirth:

“Instead of holding my baby in my arms that day, I was praying to God that I was going to make it out alive. Instead of owning up to their catastrophic mistakes, conducting tests, and finding solutions for the debilitating physical pain they caused, the hospital sent a social worker to evaluate my mental state and ask if I had diapers at home. I felt unheard, ignored, and humiliated”. [Participant #2]

Participants believed that providers were able to display a lack of accountability for their errors because “no one was watching” and they perceived they could get away with it due to participants' Black race, even when participants had other types of privilege like high education and incomes or had strong advocates. This further reinforced the racialization of medical errors and “near misses” experienced by participants because they perceived there to be no other possible contributing factors leading to their maltreatment and outcomes:

“I relate my experience repeatedly to police brutality and an abuse of power by police in that before police wore body cameras, their story was the story. A doctor or a nurse is no different. The exception though is doctors and nurses are not wearing body cameras. When they take you into the operating room, there are no other eyes witnessing what is or is not happening. What is or is not being injected into your body. There is no one else writing down in an operative report or a chart note, what did or did not happen, very similar to a police report. So, whatever it is they say happened, that is what happened. And you bear the burden of proving something different. And that for me is not justice. That is a faulty system working against the average human being”. [Participant #4]

The “Medical Error and Near Miss theme” encompasses both *medical errors and accountability*, highlighting not only the errors themselves but also the lack of accountability in acknowledging these errors. It is important to note that our findings on medical errors and lack of documentation are based on participants' self‐reported accounts alone, as this study did not include review of medical records. Nonetheless, these themes emerged through our analyses, reflecting participants' lived experiences of care.

## 4 DISCUSSION

In our qualitative evaluation of Black women with severe maternal morbidity (SMM) in high maternal mortality settings, we identified several critical factors shaping experiences of racism during obstetric care. As efforts escalate to combat racism and improve maternal health outcomes, it becomes imperative to center the lived experiences of Black women. Our findings offer tangible instances of racism as it manifests in childbirth; participants' narratives can be used to guide recommendations aimed at combating obstetric racism within care systems.

Participants faced diverse acute SMM diagnoses, with emergent issues often overlooked by medical personnel. Similar to other studies, concerns were downplayed or disregarded, emphasizing the necessity for active listening, especially during emergencies. Some SMM cases could have been prevented with timely attention, highlighting the urgency for improved patient–provider communication and health system responsiveness. Current hospital abstraction measures often miss CDC‐defined SMM cases, emphasizing the need for a more inclusive reporting system.

Our research contributes to a growing body of evidence that suggests obstetric care for Black women is marred by systemic inequities. Studies across various geographical regions and healthcare settings have similarly reported instances of communication failures, diagnostic lapses, differential treatment, and clinical errors, highlighting a global issue of racial bias and discrimination in maternity care. By situating our findings withing this broader context, we emphasize the universal challenge of combating racism in healthcare and the urgent need for widespread change.

While our participants were predominantly highly educated with high incomes, self‐advocacy alone proved insufficient against obstetric racism. Even with socioeconomic privilege, participants encountered dismissals and ignorance of concerns. This challenges assumptions that high education and income protect against obstetric racism. The reality remains that higher income and education levels are not shields from the impacts of obstetric racism. This contradicts common assumptions that socioeconomic status can serve as a protective factor against healthcare disparities. It suggests that the forces of racism and discrimination operate independently of, and perhaps even compounded by, other social determinants of health. This insight calls for a deeper examination of how healthcare systems can better serve all patients, regardless of their socioeconomic background, and underscores the need for healthcare providers to adopt a more holistic and equitable approach to care. A systems‐based approach, integrating patient and provider feedback, is suggested. Interventions fostering patient advocacy and communication have shown success in improving health outcomes among marginalized populations, potentially adaptable to obstetric settings.

Limitations include a modest sample size, but the rich qualitative data compensate for quantity. Survivorship bias may influence results, and future studies involving family survivors could provide additional insights. Geographical variations could yield different findings, emphasizing the importance of considering diverse demographic profiles and racial disparities.

### 4.1 Conclusions

In conclusion, SMM experiences among Black women underscore the impact of racism, revealing instances of communication failures, stereotyping, differential treatment, and medical errors. Even strong self‐advocates faced dismissals during emergencies. To reduce racism and enhance maternal health, interventions must prioritize Black women's experiences, emphasizing improved patient–provider communication and competent, responsive healthcare systems.

## FUNDING INFORMATION

This work was funded by the Georgetown University Gender and Justice Initiative Fellowship (awarded to W.P.).

## CONFLICT OF INTEREST STATEMENT

None.

## Data Availability

The data that support the findings of this study are openly available in ORCID at https://orcid.org/my‐orcid?orcid=0000‐0003‐2705‐3279, reference number 0000‐0003‐2705‐3279.
